# Automatic Brain Categorization of Discrete Auditory Emotion Expressions

**DOI:** 10.1007/s10548-023-00983-8

**Published:** 2023-08-28

**Authors:** Siddharth Talwar, Francesca M. Barbero, Roberta P. Calce, Olivier Collignon

**Affiliations:** 1 Institute for Research in Psychology (IPSY) & Neuroscience (IoNS), Louvain Bionics, University of Louvain (UCLouvain), Louvain, Belgium; 2 School of Health Sciences, HES-SO Valais-Wallis, The Sense Innovation and Research Center, Lausanne and Sion, Switzerland

**Keywords:** Emotion, Voice, Categories, EEG, Frequency tagging

## Abstract

Seamlessly extracting emotional information from voices is crucial for efficient interpersonal communication. However, it remains unclear how the brain categorizes vocal expressions of emotion beyond the processing of their acoustic features. In our study, we developed a new approach combining electroencephalographic recordings (EEG) in humans with a frequency-tagging paradigm to ‘tag’ automatic neural responses to specific categories of emotion expressions. Participants were presented with a periodic stream of heterogeneous non-verbal emotional vocalizations belonging to five emotion categories: anger, disgust, fear, happiness and sadness at 2.5 Hz (stimuli length of 350 ms with a 50 ms silent gap between stimuli). Importantly, unknown to the participant, a specific emotion category appeared at a target presentation rate of 0.83 Hz that would elicit an additional response in the EEG spectrum only if the brain *discriminates* the target emotion category from other emotion categories and *generalizes* across heterogeneous exemplars of the target emotion category. Stimuli were matched across emotion categories for harmonicity-to-noise ratio, spectral center of gravity and pitch. Additionally, participants were presented with a scrambled version of the stimuli with identical spectral content and periodicity but disrupted intelligibility. Both types of sequences had comparable envelopes and early auditory peripheral processing computed via the simulation of the cochlear response. We observed that in addition to the responses at the general presentation frequency (2.5 Hz) in both intact and scrambled sequences, a greater peak in the EEG spectrum at the target emotion presentation rate (0.83 Hz) and its harmonics emerged in the intact sequence in comparison to the scrambled sequence. The greater response at the target frequency in the intact sequence, together with our stimuli matching procedure, suggest that the categorical brain response elicited by a specific emotion is at least partially independent from the low-level acoustic features of the sounds. Moreover, responses at the fearful and happy vocalizations presentation rates elicited different topographies and different temporal dynamics, suggesting that different discrete emotions are represented differently in the brain. Our paradigm revealed the brain’s ability to automatically categorize non-verbal vocal emotion expressions objectively (at a predefined frequency of interest), behavior-free, rapidly (in few minutes of recording time) and robustly (with a high signal-to-noise ratio), making it a useful tool to study vocal emotion processing and auditory categorization in general and in populations where behavioral assessments are more challenging.

**Supplementary Information:**

The online version contains supplementary material available at 10.1007/s10548-023-00983-8.

## Introduction

In humans and other animals, efficient categorization of emotion expressions is crucial for effective social interactions and survival. In Darwin’s milestone book “*The Expression of the Emotions in Man and Animals”* published in 1872, attention was mostly focused on the face as the carrier of emotion expressions. Since then, research on facial emotion expressions has led to the suggestion that at least six basic emotions- anger, disgust, fear, happiness, surprise and sadness (Ekman 1993) can be expressed using specific facial movements as coded by the Facial Acting Coding System (FACS, Ekman & Friesen 1978; Waller et al. 2020). These emotion categories are expressed similarly across different cultures (Elfenbein & Ambady 2002), arise very early in development (Flom & Bahrick 2007; Poncet et al. 2022), and can be found in our evolutionary ancestors (Darwin 1872; Waller & Micheletta 2013).

Although Darwin mentioned the importance of vocalizations as a carrier of affective signals (Darwin 1872), it was suggested that voice was yet to be proven an effective source for detecting discrete emotion categories (Ekman 2009). However, mounting evidence shows that discrete emotion expressions can be delivered and decoded through vocal expressions with a high accuracy in humans (Cornew et al. 2010; Falagiarda and Collignon 2019; Pell and Kotz 2011). In fact, the same discrete emotion categories as those found for faces (Ekman 1993) also express similarly through voices across cultures (Juslin and Laukka 2003; Sauter et al. 2010; Sauter and Eimer 2010; Scherer et al. 2001) and emotion detection from the voice also develops early in infancy (Izard et al. 1980; Mehler et al. 1978; Zhao et al. 2021).

It is well known that the distinct acoustic features that characterize different discrete categories of vocal emotions activate different patches in the core region of the auditory cortex that is known to be tonotopically organized (Talavage et al. 2004). However, functional magnetic resonance imaging (fMRI) studies revealed that some temporal regions show distinct activations for diverse affective vocalizations (Frühholz and Grandjean 2013b; Ethofer et al. 2012), partially independent of acoustic amplitude and frequency cues (Giordano et al. 2021; Grandjean et al. 2005). In addition to auditory cortices, other brain regions are commonly activated across a wide range of auditory emotion categories, such as the amygdala (Fecteau et al. 2007; Wiethoff et al. 2009) and the medial prefrontal cortex (Etkin et al. 2011; Kober et al. 2008). Despite the existence of these regions involved in the processing of various emotion expressions, it is still debated whether discrete emotions may recruit separate brain areas (Calder et al. 2001; Ethofer et al. 2009; Frühholz and Grandjean 2013a; Johnstone et al. 2006; Kober et al. 2008; Kotz et al. 2013; Mauchand & Zhang 2022; Phan et al. 2002; Phillips et al. 1998; Vytal and Hamann 2010).

Affective vocalizations have been extensively studied using event related potentials (ERPs). Components as early as ~ 100 ms which usually reflect acoustic processing were found to be modulated by emotional non-speech utterances compared to neutral vocalizations (Jessen et al. 2012) This may suggest that the difference in early ERPs are driven by acoustic features (Salvia et al. 2014). In addition, there is also evidence of enhancement of later ERP components such as the early posterior negativity (EPN: 200–350 ms) and late positive potential (LPP: ~ 400 ms) when emotional utterances are contrasted with neutral vocalizations (Frühholz et al. 2011; Jessen and Kotz 2011). These later differences are thought to index the mechanism of affective categorization (Schirmer and Kotz 2006) that may be partially independent from acoustic differences. However in most electrophysiological studies, the responses to emotional vocalizations are compared to neutral vocalizations rather than to different emotions, thus leaving unanswered whether the observed differences are due to acoustic differences (Banse and Scherer 1996; but see Bostanov and Kotchoubey 2004; Pell et al. 2015) or change in valence or arousal rather than the categorisation of discrete emotion expressions.

In this study, we aimed to develop a novel approach combining electroencephalographic recordings (EEG) in humans with a frequency-tagging paradigm to provide an objective measure of automatic categorization of vocal emotion expressions beyond the processing of acoustic features. The frequency-tagging approach (Regan 1989) relies on the fact that under external stimulation of periodic stimuli, the brain regions that encode the stimuli synchronize at the exact same frequency (Norcia et al. 2015). We adapted the Fast Periodic Auditory Stimulation paradigm (FPAS, Barbero et al. 2021) to present different exemplars of discrete auditory emotion categories. The stimuli were presented periodically at 2.5 Hz (e.g. the stimulus length is 350 ms with a 50 ms silence/gap). Importantly, within the sequence of affective vocalizations, there is also embedded another periodically occurring target emotion category (e.g. different exemplars of Fear presented at every third position at 0.83 Hz). Thus, the brain will elicit a response at the general rate of sound presentation and its harmonics (2.5 Hz, 5 Hz, 7.5 Hz etc.) if it can segregate different affective sounds. Crucially, we will be able to observe a response at the target frequency and its harmonics (0.83 Hz, 1.66 Hz, 3.32 Hz etc.) only if the participants’ brain can *discriminate* the vocalization of the target category from sounds of other frequent non-target categories as well as *generalize* all target vocalizations to one common emotion category (see Barbero et al. 2021).

From a fundamental point of view, our study aimed to investigate whether the human brain categorizes discrete auditory emotion expressions partially independently from their acoustic properties. This was implemented by the careful selection of non-verbal vocalizations with similar acoustic properties (spectral center of gravity, harmonicity-to-noise ratio, pitch) and introducing a second control stimulation sequence of frequency-scrambled sounds with similar spectro-temporal profile (frequency content, sound’s envelope) to the intact sounds and identical periodic constraints but disrupted intelligibility (Barbero et al. 2021; Dormal et al. 2018). In addition to the frequency domain analysis, we conducted time-locked analyses to investigate the time course of the response underlying emotion categorization. Finally, our study also aimed to provide a powerful tool to investigate the brain’s ability to categorize auditory emotion expressions objectively (the response lies at predefined frequencies of interest), robustly (with a high signal-to-noise ratio), and automatically (does not need an explicit request to process affective vocalizations).

## Materials and Methods

### EEG Experiment

#### Participants

Twenty-four participants (12 females, mean age: 22.29, S.D.: 2.33, range: 19–28 years) participated in the study. All participants reported no history of neurological or audiological disorders and were right-handed. Auditory deficits were self-reported by the participants since there are strong associations of self-reported and audiometric hearing loss, especially within the age range of our sample set (Gomez et al. 2001; Hannula et al. 2011; Kiely et al. 2012). The experiment was approved by the local ethical committee of the Catholic University of Louvain (UCLouvain, project 2016–25). All participants provided written informed consent and received financial compensation for their participation.

#### Stimuli

**Fig. 1:**
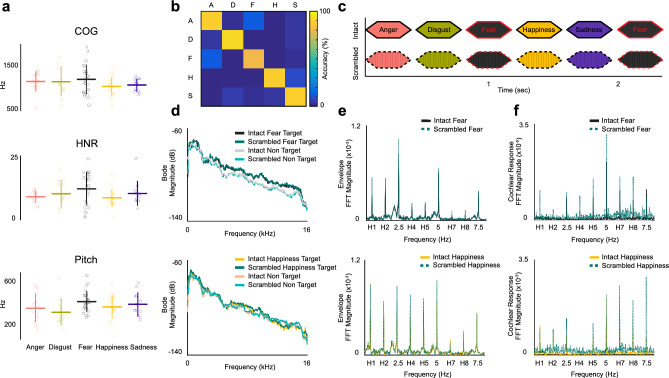
Stimuli and sequences: **A** The selected sounds of five emotion categories have similar spectral properties: center of gravity (COG), harmonicity to noise ratio (HNR) and pitch. **B** Behavioral experiment validated 84 short non-verbal vocalizations to depict the appropriate emotion category. A = Anger, D = Disgust, F = Fear, H = Happiness, S = Sadness. **C** Sounds were presented periodically at 2.5 Hz. The target emotion (e.g. fear in this illustration) repeated at every third position leading to a target presentation rate of 0.83 Hz while other emotion categories were presented randomly. **D** Bode plot shows similar averaged spectral power of intact and scrambled stimuli for target and non-target sounds for each emotion condition. **E** Similar averaged FFT distribution of the envelope between intact and scrambled sequences indicates similar temporal profiles across the two types of sequences in each emotion condition. **F** No significant difference found in the averaged FFT magnitudes of the simulated cochlear response to intact and scrambled sequence, verifying similar spectro-temporal profile of all sequences as well as the processing of acoustic features at the cochlear level

We selected sounds of five primary emotion categories: anger, disgust, fear, happiness and sadness. The stimuli were extracted from clips in which professional actors and actresses performed emotion expressions in varied styles and intensities without any linguistic content (Belin et al. 2008). Additionally, a few stimuli were selected from a database of non-verbal vocalizations depicting several distinct varieties of emotion categories (Cowen and Keltner 2017). A total of 96 heterogeneous sounds were cropped to a length of 350 ms to allow periodic presentation. All sounds were equalized in overall energy (root mean square, RMS) and 10 ms ramps were applied at the start and at the end of the stimuli to avoid clicking. To make sure that the empirical EEG responses to emotion vocalizations are not driven by different acoustic features across different emotion categories, we confirmed that the sounds have comparable spectral center of gravity (COG; F = 1.1882, p = 0.3224, ηp2 = 0.0567), harmonicity-to-noise ratio (HNR; F = 1.6930, p = 0.1599, ηp2 = 0.0789) and pitch (F = 1.7618, p = 0.1448, ηp2 = 0.0819) across the five emotion categories (Fig. 1a). The acoustic properties were computed using custom scripts in Praat (Boersma and Weenink 2001).

To select emotional stimuli eliciting a reliable recognition of the intended emotion, we conducted a behavioral experiment where 10 participants (mean age = 27.7, S.D. = 4.6200; who did not participate in the EEG experiment) categorized each sound as pertaining to one of the five selected emotion categories using a five-alternative forced choice task. 3 blocks were presented in a randomized fashion where each block was composed of 96 stimuli with 1 repetition per block (anger, disgust, sadness: 14 each; fear and happiness: 27 each). Among those sounds, 84 sounds across 5 categories were selected (anger, disgust, sadness: 12 each; fear, happiness: 24 each). All selected sounds achieved 80% or more recognition accuracy across all participants (chance level: 20%) and were subsequently used to build the sequences for the EEG session (Fig. 1b). We required double number of stimuli for Fear and Happiness categories to present an equal number of unique stimuli of each category in the sequence (More details in Sect. “Sequences and Procedure”). Each emotion category consisted of an equal number of sounds by male and female actors to incorporate heterogeneous spectro-temporal profiles across the stimuli set.

To create the control sequences, the selected stimuli were frequency-scrambled (as in Dormal et al. 2018) to preserve the overall frequency content of the original sounds while disrupting their harmonicity and intelligibility. Specifically, we extracted the sound envelope of each sound using the Hilbert transform and computed a Fast Fourier Transform (FFT) of each sound to shuffle the magnitude and phase of frequency bins within consecutive windows of 200 Hz. Then, we computed the inverse FFT and we applied the original sound envelope to the resultant scrambled waveform. This procedure led to a disruption in harmonicity and intelligibility of the scrambled sounds (as confirmed in a behavior experiment: see Sect. “Experiment 2-Behavior”), while keeping their spectro-temporal structure almost identical to the original/intact sounds (Fig. 1d, e). The overall energy (RMS) of all scrambled sounds were equalized and ramps of 10 ms were applied same as for the intact sounds.

#### Sequences and Procedure

The affective sounds were placed one after the other in a periodic fashion with an inter-stimulus interval (ISI) of 50 ms to facilitate sound segregation and discrimination. Thus, the general rate of presentation or base rate was 2.5 Hz (i.e., 1/(0.350 + 0.050) s). Importantly, each third sound in the sequence belonged to a specific emotion category which was presented periodically at a target frequency of 0.833 Hz (2.5 Hz/ 3), while all sounds of non-target categories were presented non-periodically. The non-target emotion categories were presented once before being repeated. Two emotion conditions were created, where Fear and Happiness were used as the target category, respectively. Fear was chosen due to its salience and evolutionary function (Stanley 1984). Happiness was selected due to its different valence amongst the other chosen emotions with negative valence. For each emotion condition, unique sequences were constructed such that the order of stimuli in each sequence was randomized to increase generalizability. The order of stimuli presentation of each frequency intact sequence was replicated in a corresponding frequency-scrambled sequence. As a result, each intact sequence had an identically ordered control scrambled sequence (Fig. 1c). With 24 unique targets of 1 emotion category (Fear or Happiness) and 48 unique non-targets across the other 4 emotion categories in any sequence, each sound was presented twice in a sequence (total sounds: 144), leading to a sequence length of 57.6 s. Each sequence included 2 s of fade-in and fade-out where the volume gradually increased from 0 to maximum amplitude of the sounds at the start and vice-versa at the end of the sequence, in order to avoid abrupt movements of the participants at the onset of each sequence which could have introduced artifacts in the recordings.

Therefore, four different conditions were presented: two different sequence conditions (intact sequence with conditions: Fear and Happiness as target) and to control for low-level acoustic confounds (scrambled frequency sequences), namely Fear Intact, Fear Scrambled, Happiness Intact, Happiness Scrambled. Ten instances of each sequence group (total: 40) were created prior to the EEG session and presented to each participant in a pseudo-randomized fashion. The speakers were placed at 1 m distance and all sounds were presented at around 60 dB measured from the ears of the participants. The participants kept their eyes closed during sequence presentation and were instructed to press a button when they heard a sound lower in volume as compared to the other sounds. The lower volume was implemented by reducing the sounds’ root mean square (RMS) value by a factor of 10. This attention target was presented 6 times per sequence in a randomized fashion, excluding the 2 s fade-in and fade-out at the start and end of the sequence. Examples of the sequences are available at: https://github.com/sid0309/freqtag_emotion. The experiment was designed on MATLAB R2016b (Mathworks) using the Psychtoolbox and extensions (Brainard 1997; Kleiner et al. 2007; Pelli 1997).

#### EEG Acquisition

During acquisition, participants sat in a dimly lit room and EEG was acquired with a Biosemi ActiveTwo System (https://www.biosemi.com/products.htm) using 128 channel Ag/AgCl electrodes. International 10–20 system were used for the recording sites as well as their intermediate positions (position coordinates can be found at https://www.biosemi.com/headcap.htm). Additionally, two surface electrodes were applied at mastoids. The acquisition sampling rate was 512 Hz. The magnitude of offset of all electrodes that were referenced to the common mode sense (CMS) were kept below ± 25.

#### Analysis

Data was analyzed using Letswave 6 (https://github.com/NOCIONS/Letswave6) and the Fieldtrip toolbox (Oostenveld et al. 2011) running on MATLAB R2016b (Mathworks) with custom built scripts in MATLAB and on Rstudio (Rstudio Team 2020). Subsequent sections of data preprocessing and the frequency domain analysis followed a pipeline that has been adopted in many frequency-tagging studies in vision (Bottari et al. 2020; Dzhelyova et al. 2017; Retter and Rossion 2016; Rossion et al. 2015, 2020; Volfart et al. 2021) and audition (Barbero et al. 2021).

##### Preprocessing

Raw continuous EEG data was filtered using a fourth order Butterworth band-pass filter from 0.1 to 100 Hz and a notch filter at 50 and 100 Hz with a width of 0.5 Hz to attenuate the power line noise. The data was then down-sampled to 256 Hz to facilitate data handling and storage. Continuous data was then segmented from 2 s before the onset of the sequences to 2 s after the end of the sequence, resulting in a trial length of 61.6 s. Subsequently, the ICA matrix of each segmented trial was computed using RUNICA (Makeig et al. 1995) and the resulting components for each participant were visually inspected. Artefactual frontal components due to facial movements, when present, were deleted with at most one component deleted in 18 out of the 24 participants. Visual inspection was then conducted to detect potential noisy trials and/or electrodes. Trials containing residual artefactual activity were deleted (on average 1.6 trials out of the 40 trials per participants, maximum number of trials deleted was 5 in two participants). Noisy channels were linearly interpolated using the three closest neighboring electrodes (2 channels for 5 subjects—FT8, FC5; T8h, AF7; FPz, AFF2; AF3, P9; PPO5, AFF2 respectively—and 1 channel for 4 other subjects—I1; I1; C6; CPP5h). The trials were subsequently re-referenced to the average reference and divided into two emotion conditions (Fear and Happiness) and its two sequence types (Intact and Scrambled): Fear Intact, Fear Scrambled, Happiness Intact and Happiness Scrambled.

##### Frequency Domain Analysis

Considering the presentation rate of the target emotion category and the frequency resolution (1/duration of the sequence), all trials were re-segmented from 2 s after sequence onset (to remove fade-in) to a length of 52.8 s, to contain an integer number of target presentation cycles (0.833 Hz). Trials were then averaged in the time domain separately by emotion condition (Fear and Happiness) and sequence type (Intact and Scrambled) to attenuate the noise and brain responses that were not time-locked to the stimuli (Luck 2014). The resulting averaged trials for the four conditions were then grand-averaged across participants. Consequently, a Fast Fourier Transform (FFT) was applied to the averaged trials. Amplitude spectra extended from 0 to 128 Hz with a frequency resolution of 0.0189 Hz, allowing us to isolate the base response (general presentation rate) at 2.5 Hz and target response at 0.833 Hz, along with their respective harmonics. The harmonic responses in addition to fundamental frequency (in this case 2.5 Hz or 0.83 Hz) can be accounted for in relation to complex responses of the brain, corresponding to the principles of frequency domain analysis of periodic signals (Retter et al. 2021). Since the summation of harmonics in the FFT can indicate the overall responses in single values (Retter et al. 2021; Retter and Rossion 2016), we applied a criterion to select significant harmonics to include in further analysis. Significance of harmonics was determined by first pooling all 128 channels together and computing the z-score at each frequency bin, considering 12 bins at each side of the frequency bin of interest, excluding the immediate adjacent bins and the maximum and minimum of the entire window (Retter and Rossion 2016). For each target emotion condition (Fear and Happiness) and sequence type (Intact and Scrambled), consecutive harmonics that displayed a z-score > 2.32 (p < 0.01, 1-tail, *signal* > *noise*) were considered significant. While considering significant harmonics for the target emotion conditions, the base frequency bins (i.e., 2.5, 5, 7.5 Hz etc.) were not taken into account. The chosen number of consecutive harmonics for Intact and Scrambled sequences were equalized by considering the highest number of significant consecutive harmonics in any of the two types of sequences, knowing that adding responses at non-significant harmonics is not detrimental for the calculation of the response (i.e., adding zeros, Retter et al., 2018). To quantify the responses at base and target frequencies, we computed the baseline subtracted amplitudes on the grand-averaged FFT of each condition and type, where the window for this calculation was defined similarly to the computation of z-scores (12 bins on either side except maxima, minima and adjacent bins). Baseline subtraction takes into account the different noise profiles across different frequency bands (Luck 2014), especially higher noise at low frequencies (< 1 Hz) in EEG recordings. Consequently, we summed the baseline subtracted amplitude at the selected significant harmonics to finally represent them as topographical maps. For illustration purpose, the signal-to-noise ratio (SNR) was computed using the same criteria to estimate the noise (12 bins on either side, excluding the immediate bins on either side, the maximum and minimum).

To find the electrode(s) of interest involved in emotion categorization for Fear and Happiness that could be independent of low-level acoustic processes, we subtracted the grand averaged FFT spectra of the frequency-intact and frequency-scrambled sequences (*intact—scrambled*) for each target emotion condition separately. Then, we extracted chunks of the resultant spectra, where each chunk consisted of 25 frequency bins (central bin of the chosen harmonic and 12 neighboring bins on each side). The number of chunks was defined as the number of chosen harmonics in each emotion condition. The sum of the chunks and then, the z-score were computed at the harmonic (13th bin) for each channel (Volfart et al. 2021). Finally, we performed FDR (Benjamini and Hochberg 1995) correction across 128 channels for multiple comparisons, for each emotion condition. Additionally, to demonstrate the response of each individual and consider inter-individual variability, we repeated the analysis by applying the FFT to the average of all trials of each condition, type and participant. Baseline subtracted amplitudes were calculated at the target frequency and its significant harmonics (that were found as per the grand-averaged analysis for each emotion condition) and consequently summed.

##### Time Domain Analysis

We conducted time-locked analysis to characterize the time course of emotion discrimination process. The raw data was filtered through the Butterworth bandpass and the notch filter with the same parameters as in frequency domain analysis. An additional notch filter was applied to the filtered data to remove activity related to the sound presentation rate (at 2.5, 5, 7.5 Hz) which would reflect general auditory processes not linked to emotion discrimination. Stimulus-locked epochs of 800 ms ranging from the onset of one stimulus prior to the target stimulus to the end of the target stimulus were extracted such that each sequence yielded 44 epochs. The first 2 and last 2 epochs were deleted to exclude the fade in and out of the sequences. Noisy epochs with amplitude deflections exceeding ± 100 µV in any channel were deleted. The resultant epochs were re-referenced to averaged mastoids, then equalized in number across conditions and types separately and averaged for each subject. Baseline correction was implemented by subtracting the signal within 400 ms pre-stimulus activity, corresponding to 1 cycle of base rate, i.e. the prior epoch to the target epochs (Dzhelyova et al. 2017). Finally, the baseline subtracted epochs were grand-averaged across all subjects for each condition and type separately.

To compare the temporal activity of emotion categorization, we conducted a time point-by-time point, one-tailed t-test between frequency intact and scrambled sequences across subjects for each electrode and emotion condition. FDR correction was applied across 128 channels to correct for multiple comparisons (Benjamini and Hochberg 1995). Segments of data were considered significantly different if the two conditions (intact vs scrambled) were different for more than 25 ms, i.e. > 13 consecutive time-points (Chen et al. 2021).

### Cochlear Model and Envelope

To ensure that potential differences in the EEG responses elicited by different conditions could not be merely explained by differences in the temporal structure of the target emotion categories of interest, we compared the temporal envelopes of intact and scrambled sequences. We prepared unique sequences for each participant before the EEG session (40 per individual) and extracted the envelope of each sequence using the Hilbert Transform. Then, we computed the FFT of all envelopes and averaged them across each sequence type (intact and scrambled) and condition (Fear and Happiness) for each participant. Further, we chose the harmonics of interest in the FFT based on the empirical EEG data (see Sect. “Frequency Domain Analysis” for the procedure and Sect. “Frequency Domain: Base Response” for EEG results) and summed the FFT magnitudes across the chosen bins for every subject. Finally, a 1-tailed t-test was calculated across all participants to contrast intact from scrambled sequences. Additionally, to assess how spectral and temporal characteristics of sound alone can influence early responses processed in the cochlea, we employed gammatone filters using Auditory Toolbox (Slaney 1994) to simulate a cochlear response to a given sequence. Cochlear simulation was computed on each sequence before the EEG session. The FFT was applied on the simulated cochlear response and each sequence type (intact and scrambled) and condition (Fear and Happiness). Then, we chose the harmonics of interest in the FFT of the cochlear response based on the empirical EEG data (see Sect. “Frequency Domain Analysis” for the procedure and Sect. “Frequency Domain: Base Response” for EEG results) and summed the FFT magnitudes across the identified bins of interest for each subject. Lastly, we compared the summed FFT magnitudes of intact and scrambled sequences across all participants using a 1-tailed t-test (*intact* > *scrambled*) for each condition separately.

### Behavioral Experiment

We conducted a behavioral experiment to evaluate whether the participants could efficiently identify emotion from a stream of short bursts of affective voices, classify vocalizations to discrete emotion categories and to confirm the unintelligibility of the scrambled sounds.

#### Participants

21 out of 24 participants (11 women, age: 22.28, SD: 2.36, range: 19–28 years) who took part in the EEG experiment participated in the behavioral experiment. This session was scheduled after the EEG experiment to avoid familiarity with the sounds during the EEG session. All participants reported no history of neurological or audiological disorders and were all right-handed. The experiment was approved by the local ethical committee of Catholic University of Louvain (UCLouvain, project 2016–25). All participants provided written informed consent and received financial compensation for their participation.

#### Procedure

Stimuli from the EEG session were used in the behavioral experiment (see Sect. “Stimuli”). The behavioral session was divided into two tasks: (1) a “Sequence task” where subjects were asked to identify a target emotion category amongst sounds presented in a short sequence and (2) an “Isolation task”, where each sound was presented separately. The sequence task was divided into four blocks, each block consisting of 48 short sequences and each sequence comprising of five emotional sounds. Two of the four blocks required the participants to identify if a Fearful vocalization was present amongst the sounds presented while in the other two blocks, participants were instructed to detect whether a Happy vocalization was present in the sequence. To mimic the structure of the sequences used in EEG experiment, we inserted the target emotion at the third place in each short sequence. Subjects were asked to perform a two-alternative forced-choice task (target emotion present or not) with half of the sequences in each block (24/48) consisting of the target emotion category (Fear or Happiness according to the block). All sequences and blocks were presented in a random order. Later, participants performed the “Isolation task” which was divided into four blocks, each consisting of 84 sounds. In two blocks, subjects were asked to listen to a single affective vocalization and answer three questions- (a) classify the sound to one of the five emotion categories: anger, disgust, fear, happiness or sadness in a five-alternatives forced choice task; (b) rate the valence of each sound on a scale from 1 to 5 (1 = most negative, 3 = neutral, 5 = most positive); (c) rate the arousal evoked by the stimuli on a scale from 1 to 5 (1 = not aroused at all, 5 = most aroused). In the other two blocks, the scrambled version of the same sounds were presented and the subjects were asked to classify them to an emotion category in a five-alternatives forced choice task. All sounds and blocks were presented in a randomized order. The experiment was implemented on Psychtoolbox and extensions (Brainard 1997; Kleiner et al. 2007; Pelli 1997) running on MATLAB R2016b (Mathworks). For analysis, sensitivity indices were calculated for each task using D-primes (d’) constituting an unbiased quantification of performance in detection tasks considering both hits and false alarms (Hautus et al. 2021; Tanner and Swets 1954).

## Results

### Experiment 1- EEG

#### Frequency Domain: Target Response

**Fig. 2:**
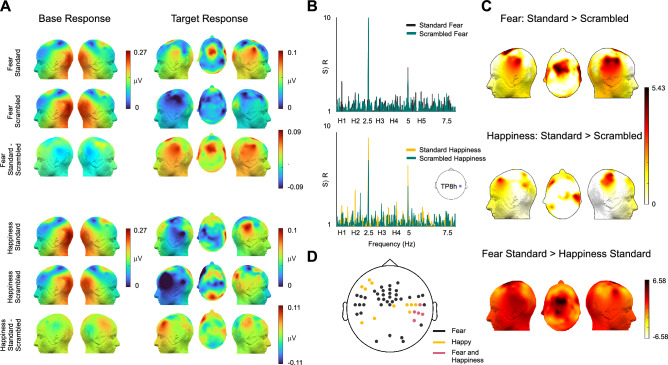
EEG results: **A** Sum of baseline subtracted amplitudes are represented as topographies. The base response appears similar across all conditions and types. The target response to Intact Fear is localized to bilateral temporal and central areas while response to Intact Happiness is elicited in the right temporal and left frontal region. Higher responses observed for intact stimuli sequences than scrambled stimuli sequences. **B** Signal to noise ratio (SNR) at channel TP8h for both emotion conditions to visualize the response across frequencies. H1 and H*n* refer to the first harmonic of target frequency i.e. 0.83 Hz and higher harmonics, respectively. **C** Scalp regions eliciting significant emotion-selective responses Fear is significant at bilateral temporal and central regions; Happiness at right temporal and left frontal areas. Channels in central and bilateral temporal regions are more selective to Fear than Happiness while no channels are more selective to Happiness. **D** Distinct, yet overlapping channels selective to Fear and Happiness

The target response was quantified as the sum of consecutive significant harmonics, i.e., 5 harmonics for Fear (0.833, 1.666, 3.333, 4.166 and 5.83 Hz) and 4 for Happiness (0.833, 1.666, 3.333 and 4.166 Hz). Presence of a response in each condition and type was assessed by computing the z-scores in all channels. (*Fear Intact* > *0*: 107 channels, maximum at TP8: z = 10.2913, p = 3.8550 × 10^–25^; *Fear Scrambled* > *0*: 77 channels, maximum at PO10: z = 6.3015, p = 1.4731 × 10^–10^; *Happiness Intact* > *0*: 110 channels, maximum at TP8: z = 10.0758, p = 3.5342 × 10^–24^; *Happiness Scrambled* > *0*: 100 channels, maximum at PPO6: z = 9.7109, p = 1.3553 × 10^–10^, all p-values are FDR corrected). Crucially, after pooling all channels in each condition separately, the sum of baseline subtracted amplitudes at the harmonic bins of interest revealed a higher amplitude for intact sequences type in comparison to scrambled for both Fear and Happiness conditions (*Fear Intact*: mean = 0.0484 μV, S.D. = 0.0613; *Fear Scrambled*: mean = 0.0292 μV, S.D. = 0.0333; *Happiness Intact*: mean = 0.0561 μV, S.D. = 0.0516; *Happiness Scrambled*: mean = 0.0379 μV, S.D. = 0.0357; topoplots depicted in Fig. 2a).

To find the channels contributing to the emotion-selective EEG responses at the target frequency and harmonics, we first computed the difference between the grand averaged Fast Fourier spectra of frequency intact and scrambled conditions at each electrode and computed the z-score for the 2 conditions: Fear and Happiness (see methods Sect. “Frequency Domain Analysis”). We found 42 significant channels for Fear selective response in bilateral temporal areas (Fig. 2c; *Fear intact—Fear scrambled* > *0*: maximum in right temporal FT8h: z = 5.3219, p = 5.1345 × 10^–8^; in left temporal TP7h: z = 4.7200, p = 1.1792 × 10^–6^) and fronto-central electrodes (maximum at FFC3: z = 5.4294, p = 2.8272 × 10^–8^). 16 significant channels for Happiness condition were clustered in right temporal (*Happiness intact – Happiness scrambled* > *0*: maximum at C6h: z = 4.8022, p = 7.8466 × 10^–7^) and left frontal area (maximum at AFF5: z = 4.3399, p = 7.1274 × 10^–6^). All p-values are FDR corrected and other significant channels with their z-scores are tabulated in Table S1 in supplementary material.

We found five significant overlapping channels across Fear and Happiness conditions over right temporal electrodes, that may indicate elicitation from regions common to emotion processing, regardless of the category: P6, TP8h, TP8, CP6 and T8h (Fig. 2d). For visualization, the signal-to-noise ratio (SNR) at the right temporal channel TP8h (significant in both Fear and Happiness conditions) shows higher SNR at the harmonics of interest for intact in comparison to scrambled (Fig. 2b). However, we also observed different clusters of significant electrodes across conditions. To investigate further whether some channels were more selective to Fear than Happiness or vice versa, we subtracted the FFT of Happiness intact from Fear intact and computed the z-score at the bins of interest. Since we previously selected different number of harmonics to quantify Fear (5 harmonics) and Happiness (4 harmonics) responses, we equalized the number of harmonics to the maximum (i.e. 5 harmonics) since adding responses at non-significant harmonics is not detrimental for the calculation of the response (i.e., adding zeros, Retter et al., 2018). The two-sided FDR corrected p-values from the z-test revealed 38 significant channels that were more selective to Fear than Happiness (Fig. 2c) mostly clustered at and around the central region (*Fear intact—Happiness intact* > *0*: maximum at FFC1: z = 6.4061, p = 7.4645 × 10^–11^). We did not find any channels significantly more selective to Happiness than Fear.

Furthermore, we also quantified each participant’s response to Fear and Happiness by averaging the summed baseline subtracted amplitude across 42 and 16 significant channels, respectively. One-tailed t-tests revealed that the contrast Intact and Scrambled is significantly superior to 0 (*Intact—scrambled* > *0*: *Fear*: mean = 0.0485 μV, S.D. = 0.0638, t_(23)_ = 3.7237, p = 5.5720 × 10^–4^, Cohen’s d = 0.7602; *Happiness*: mean = 0.0361 μV, S.D. = 0.0621, t_(23)_ = 2.8480, p = 0.0045, Cohen’s d = 0.5813, all p-values are FDR corrected). Individual baseline subtracted amplitudes are plotted in Fig S5 in supplementary material.

#### Frequency Domain: Base Response

2 consecutive significant harmonics for Fear (2.5, 5 Hz) and 3 for Happiness were identified (2.5, 5, 7.5 Hz). A similar topography was observed visually at the general rate of presentation of sounds across intact and scrambled sequences (Fig. 2a). However, z-scores revealed differences between intact and scrambled types for both Fear and Happiness emotion conditions (*Intact—Scrambled* > *0*: *Fear*: 1 channel at FC6: z = 5.2978, p = 5.8611 × 10^–8^; *Happiness*: 38 channels. maximum at FC6: z = 6.4441, p = 5.8128 × 10^–11^, p-values are FDR corrected).

#### Time Domain

**Fig. 3:**
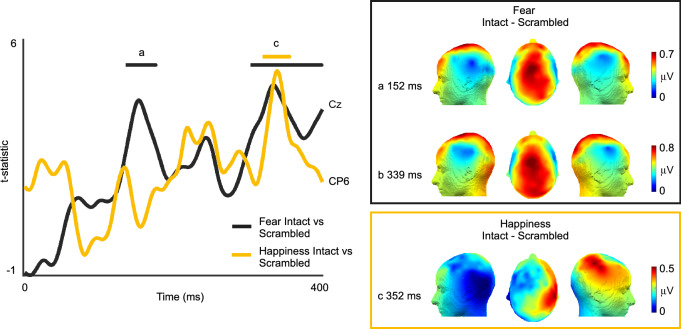
EEG time domain results: t-statistic of Fear intact > scrambled and Happiness intact > scrambled plotted with respect to time. The representation of Fear on contrasting with scrambled sequences, evolves early (peaking at 152 ms) and later (339 ms) across fronto-central-parietal channels; only one significant window for happiness was found peaking at 352 ms in the right temporal parietal region. The topographies represent the EEG amplitude at the peak time points

We compared the grand averaged trials of intact and scrambled targets with a time point-by-time point 1-tailed t-test separately for each emotion condition. The analysis revealed two significant windows for the Fear condition (Fig. 3): between 135 and 176 ms (*Intact* > *Scrambled,* peak at Cz at 152 ms: mean = 0.6780 μV, SD = 0.7978, t_(23)_ = 4.1631, p = 0.0012, Cohen’s d = 1.0257, p-values are FDR corrected) and between 311 and 398 ms (*Intact* > *Scrambled,* peak at Cz at 339 ms: mean = 0.8361 μV, SD = 0.8528, t_(23)_ = 4.6508, p = 8.8496 × 10^–4^, Cohen’s d = 0.9345, p-values are FDR corrected). These differences expressed over central, frontal and parietal electrodes. Happiness intact trials elicited a stronger response than their scrambled version across right temporal channels in only one late time window—between 319 and 356 ms (*Intact* > *Scrambled,* peak at CP6 at 337 ms: mean = 0.5764 μV, SD = 0.5628, t_(23)_ = 5.0178, p = 0.0013, Cohen’s d = 1.1390, p-values are FDR corrected) expressing over right temporal channels. Thus, in addition to different topographic representations, we also found different temporal windows of categorization of Fear and Happiness, where responses to Fear occurred earlier than Happiness. Lastly, four channels in the right temporal region: P6, TP8h, CP6 and T8h showed greater responses for intact sequences than scrambled for both Fear and Happiness conditions, consistent with the results of the frequency domain analysis.

### Cochlear Model and Envelope

After preparing the sequences of affective sounds for each subject, the FFT spectrum of the envelope of sequences of both emotion conditions (Fear and Happiness) and types (Frequency Intact and Scrambled) were averaged separately. Then, the FFT magnitude at the harmonics were summed (defined from the empirical EEG results: 5 harmonics for Fear, 4 for Happiness) and contrasted with a 1-tailed t-test suggesting similar temporal structure for both type of sequences (*Intact* > *Scrambled*: *Fear*: t_(23)_ = -0.3718, p = 0.6432, Cohen’s d = -0.1759; *Happiness*: t_(23)_ = -0.7062, p = 0.75644, Cohen’s d = -0.1442, p-values are FDR corrected across participants; Fig. 1e). Similarly, we computed a 1-tailed t-test between the summed FFT magnitudes of harmonics of interest of the simulated cochlear response to the two types of sequences (*Intact* > *Scrambled*: *Fear*: t_(23)_ = -6.9465, p = 0.9999, Cohen’s d = -1.4180; *Happiness*: t_(23)_ = − 11.7324, p = 0.9999, Cohen’s d = − 2.3949) indicating similar processing of acoustic cues at the cochlear level for both type of sequences and each emotion condition (Fig. 1f).

### Experiment 2-Behavior

**Fig. 4:**
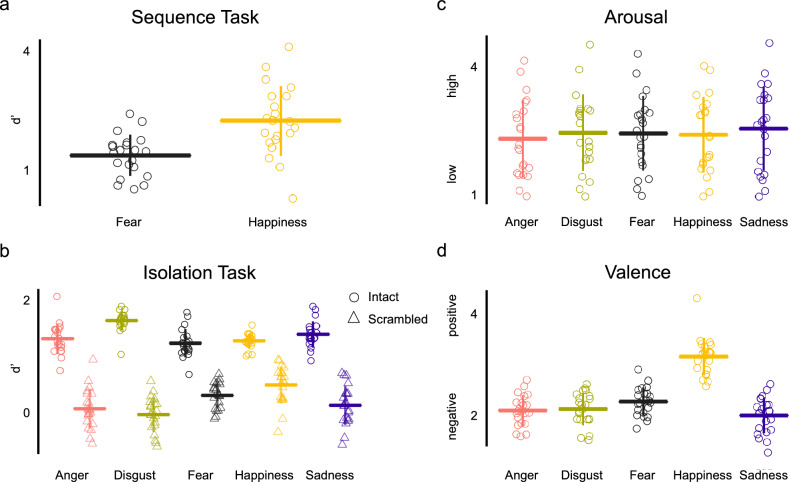
Behavioral results: **A** Sequence task: Participants could identify the presence of an exemplar of the target emotion category amongst a short sequence of sounds well above chance level. **B** Isolation task: Intact sounds were categorized to the appropriate emotion category, with worse performance for scrambled sounds, thus verifying the disruption in unintelligibility. **C** Similar arousal ratings across all emotion categories suggest no involvement of arousal in the EEG responses. **D** Happiness was the only ‘positive’ emotion amongst other categories. Greater values indicate positive valence

We conducted a separate behavioral experiment with the same participants who took part in the EEG experiment to validate:(a) if participants could categorize a short non-verbal affective vocalization played in a sequence (Sequence task), b) the unintelligibility of the scrambled sounds (Isolation task). In the Sequence task, all participants identified the presence of a target emotion amongst a stream of other emotional sounds well above chance, for both Fear (one tailed t-test *d’* > *0*: d’ values mean = 1.3866, SD = 0.5116, t_(20)_ = 12.4212, p = 7.37 × 10^–11^, Cohen’s d = 2.7103) and Happiness (one tailed t-test *d’* > *0*: d’ values mean = 2.2399, SD = 0.8638, t_(20)_ = 11.882, p = 1.61 × 10^–10^, Cohen’s d = 2.5931; Fig. 4a).

The following results in the isolation task are also tabulated in Table S2 and S3 in supplementary material. All intact stimuli were correctly categorized above chance level (Fig. 4b: one tail t-test *d’* > *0*: *Anger*: d’ values mean = 1.3214, SD = 0.2711, t_(20)_ = 22.3376, p = 1.33 × 10^–15^, Cohen’s d = 4.8742; *Disgust*: d’ values mean = 1.6475, SD = 0.1768, t_(20)_ = 42.7111, p = 0, Cohen’s d = 9.3184; *Fear*: d’ values mean = 1.2428, SD = 0.2511, t_(20)_ = 22.6799, p = 9.99 × 10^–16^, Cohen’s d = 4.9494; *Happiness*: d’ values mean = 1.2828, SD = 0.1309, t_(20)_ = 44.9205, p = 0, Cohen’s d = 9.7998; *Sadness*: d’ values mean = 1.4006, SD = 0.2331, t_(20)_ = 44.9205, p = 0, Cohen’s d = 6.0086, all p-values are FDR corrected). For scrambled stimuli, the categorization of three categories: Anger, Disgust and Sadness vocalizations were at chance level (one tail t-test *d’* > *0*: *Anger*: d’ values mean = 0.0665, SD = 0.3587, t_(20)_ = 0.8501, p = 0.4053, Cohen’s d = 0.1854; *Disgust*: d’ values mean = -0.0387, SD = 0.3132, t_(20)_ = -0.5670, p = 0.577, Cohen’s d = -0.1236; *Sadness*: d’ values mean = 0.1284, SD = 0.3523, t_(20)_ = 1.6704, p = 0.1104, Cohen’s d = 0.3645, all p-values are FDR corrected). However, d’ of scrambled versions of Fear and Happiness were found to be significantly higher than chance (one tail t-test *d’* > *0*: *Fear*: d’ values mean = 0.3071, SD = 0.2332, t_(20)_ = 6.0349, p = 6.71 × 10^–6^, Cohen’s d = 1.3169; *Happiness*: d’ values mean = 0.4956, SD = 0.3341, t_(20)_ = 6.7958, p = 1.31 × 10^–6^, Cohen’s d = 1.4834, all p-values are FDR corrected). Two explanations could be given for this result. First, there were a higher number of Fear and Happiness stimuli than the stimuli of other emotion categories, since we chose the number of stimuli based on equal presentation of each sound in a sequence (see methods Sect. “Sequences and Procedure”). Therefore, higher d’ values for Happy and Fearful vocalization could indicate an effect of training. Second, it is possible that the scrambled sounds of Fear and Happiness categories carried some amount of acoustic information which made them slightly recognizable. However, contrasting d’ of intact sounds with their frequency scrambled versions indicated that the intact vocalizations carried significantly more information than their scrambled version (two-tailed t-test *Intact* > *Scrambled*: *Fear*: SD = 0.2996, t_(20)_ = 14.306, p = 5.74 × 10^–12^, Cohen’s d = 3.12; *Happiness*: SD = 0.3841, t_(20)_ = 9.3916, p = 8.99 × 10^–9^, Cohen’s d = 2.04, all p-values are FDR corrected).

We further analyzed the arousal and valence ratings of the intact sounds. We observed similar arousal levels across all the discrete emotion categories (ANOVA F = 0.15, p = 0.9612, ηp^2^ = 0.5848; Fig. 4c) and an effect of emotion on the valence ratings ( ANOVA F = 28.15, p = 6.8228 × 10^–16^, ηp^2^ = 17.7659; Fig. 4d). As expected, post hoc t-tests revealed that valence ratings for Happiness were significantly different than other categories (*Happiness vs Anger*: p = 2.98 × 10^–8^; *Happiness vs Disgust*: p = 1.98 × 10^–8^; *Happiness vs Fear*: p = 1.81 × 10^–8^; *Happiness vs Sadness*: p = 5.96 × 10^–8^, all p-values are FDR corrected).

## Discussion

In our study, we demonstrated how combining electroencephalographic recordings (EEG) in humans with a frequency-tagging paradigm provides a robust and objective measure of a categorical brain response to short bursts of affective vocalizations. To disentangle the potential contributions of low-level auditory processing of affective vocalizations to representations of a higher order emotion categorization process, we implemented a careful selection of affective vocalizations to match their spectro-temporal properties across emotion categories. In particular, we first selected stimuli with comparable spectral features: spectral center of gravity, harmonicity-to-noise ratio (HNR) and pitch, such that no emotion category was spectrally different than the others (Fig. 1a). These sounds could be accurately classified to a discrete category of emotion: anger, disgust, fear, happiness and sadness, validated with a behavioral experiment (Fig. 1b). In addition to the periodic sequence created with intact sounds, we introduced a sound sequence with identical periodic constraints like the intact sequences but with frequency-scrambled sounds such that their spectro-temporal structure is similar but their intelligibility and harmonicity are disrupted (Barbero et al. 2021; Dormal et al. 2018; Fig. 1c,d,e). It is important to note that the difference in EEG responses between intact and scrambled sequences was unlikely to solely rely on differences in the harmonicity of our sounds since the intact sounds had comparable HNR across categories that were all presented in the same intact sequence (Fig. 1a). To ensure that emotion-selective EEG responses could not be explained by different temporal envelopes across emotion categories, we compared the FFT magnitude of the sequences’ envelopes of frequency-intact and frequency-scrambled sequences to find no significant difference at the frequency bins of interest (Fig. 1e). As a final validation, we also used Gammatone filters to simulate the cochlear response to intact and scrambled sequences and showed no differences at frequency bins of interest, at the level of early peripheral auditory processing (Fig. 1f). Thus, any difference in the EEG response to intact versus scrambled sequence cannot be simply explained by a cochlear simulation of acoustic response, nor by their spectral or envelope structure. Altogether, our stimuli selection procedure and control analyses assert that the observed categorical response to specific emotion expression categories is at least partially independent from low-level acoustic features and, therefore, likely reflects a higher-level categorization process.

We relied on a frequency-tagging technique to isolate the responses to emotion vocalizations objectively, with a high signal-to-noise ratio and automatically at specific known frequencies without having the participants to overtly respond to the sounds, thus avoiding the decisional and attentional processes to contaminate the EEG response (Levy et al. 2003). Unlike other ERP studies where emotion responses are compared to responses of neutral vocalizations causing confounds due to differences in acoustic features and in terms of arousal and valence, the method presented here provides a direct approach to obtain vocal emotion responses to discrete categories by including various heterogenous exemplars of each category. Although the frequency of presentation of sounds of the target emotion category (0.83 Hz) lies at the lower end of the frequency EEG spectrum which is susceptible to noise (Luck 2014), we acknowledged the trade-off between the frequency of presentation of target emotion category and presenting stimuli of sufficient length to allow correct emotion categorization (Falagiarda and Collignon 2019).

We observed a higher emotion-selective response to the intact sequences than the scrambled version at the target frequency and its harmonics (Fig. 2a, b). This could only occur if the brain was able to *discriminate* the target emotion from other emotion categories as well as *generalize* all sounds of the target emotion to one common category (Barbero et al. 2021). Nevertheless, the target response for the scrambled condition alone was significantly greater than 0. Such significant response in the scrambled condition could either be explained by some low-level properties of the sounds of an emotional category (as seen in the envelope or cochlear simulation) or that the response arises from some residual intelligibility that was retained, as indicated by the behavior experiment showing that the categorization of scrambled Fear and Happiness vocalizations were above chance level, but significantly lower than the categorization accuracy of the intact sounds (Fig. 4b). Thus, it can be concluded that the significantly stronger responses to intact affective vocalizations when compared to scrambled sequences (Fig. 2c, d), in conjunction with controlling of low-level features of the sounds indicate that the emotion-selective EEG response is at least partially independent of low-level acoustic features and characterize a higher-order categorization process. We also observed a robust response and a similar scalp topography at the general rate of presentation of all stimuli for both intact and scrambled sequences. However, we found a higher response to intact sequences than scrambled that could be accounted for by the overlap of the first harmonic of the general rate of presentation and third harmonic of the target response. Additionally, the higher response can be explained by a perceptual advantage of the intact sounds that may trigger enhanced attention to the stimulation.

We tested two types of sequences with either Fear or Happiness as the target emotion category repeated periodically at every third position (unknown to the participant). They expressed different topographies with part of their response spanning across few common right temporal channels suggesting the involvement of distinct yet overlapping neural substrates in emotion processing (Hamann 2012; Johnstone et al. 2006; Mauchand and Zhang 2022; Phan et al. 2002; Phillips et al. 1998). The distribution of the response to the Fear category suggests the involvement of fronto-central and temporal areas, in line with neuroimaging studies indicating large scale networks involved in the processing of fearful expressions (Kober et al. 2008; Zhou et al. 2021). Further, the different scalp topographies found for Fear and Happiness suggest different generators might be involved in the processing of the two different emotion expressions. In fact, previous research in visual emotion discrimination using Fast Periodic Visual Stimulation (FPVS) found a differential topography of the responses to distinct facial emotion expressions (Dzhelyova et al. 2017; Leleu et al. 2018; Poncet et al. 2019). Further studies are required to validate the claim by exploring the EEG responses to other emotion categories such as anger, disgust and sadness. Although the arousal ratings of all discrete emotions were similar in our study, a possible reason for different topographies may be due to the difference in valence between Fear and Happiness (Fig. 4d; Happiness has a positive valence). However, valence does not seem to be a strong driver since we observed a strong target response when Fear is the target category and Fear was not deviant in terms of valence when compared to the other emotion categories presented in the sequence.

We also investigated the time-course of the vocal categorization process. Both emotions elicited a significant response 300 ms post stimulus presentation (*Intact* > *Scrambled*), in line with electrophysiological studies that observed the late positive component (LPP) to emotional utterances (Frühholz et al. 2011; Jessen and Kotz 2011). Additionally, we observed the categorical response to Fear as early as 135–175 ms post stimulus presentation. While previous literature has reported P200 time window to be modulated by emotional interjections and non-verbal vocalizations (Charest et al. 2009; Jessen and Kotz 2011; Sauter and Eimer 2010; Schirmer et al. 2013), it is debated whether these modulations are linked to the categorical nature of emotion expressions or to differences in arousal (Paulmann et al. 2013). But due to comparable affective dimensions (valence and arousal) of Fear with other categories in our stimuli sequence (Fig. 4c, d), we speculate that the early ERP evoked by various vocalizations depicting Fear could be an early marker of its categorization, supporting recent evidence of the brain’s ability to represent discrete categories as early as < 200 ms (Giordano et al. 2021). In fact, faster responses to fear may be possible due to the potential contribution of subcortical pathways (Pessoa and Adolphs 2010) and primitive circuits recognizing danger or fear which are preserved in mammals and in humans (LeDoux 2012). Further, the absence of differences between intact and scrambled sequences before 100 ms supports the idea that the EEG responses to both emotion categories are not elicited by low-level acoustic properties of the sounds, which are well known to modulate early ERP components such as the N100 (Näätänen and Picton 1987). Another interesting observation found in both frequency and time domain analysis (post 300 ms), was that the responses from four channels in the right temporal region (P6, TP8h, CP6 and T8h) were found to be significantly greater for intact sequences than scrambled for both Fear and Happiness conditions, putatively suggesting common processes for vocal emotion processing in the right temporal regions.

There are well known differences between the spectral and temporal properties between fear, happiness and other emotion categories (see Juslin and Laukka 2003; Scherer 2003). These differences are integral in disassociating one emotion category from another. For instance, studies that used morphing of affective vocalizations of various categories, hence impacting the acoustic cues of the sounds, manipulated perceived emotions (Giordano et al. 2021) or found lower reaction time to classify a morphed vocalization (Whiting et al. 2020). The debate does not lie at how these acoustic differences express over sensory regions, but at the spatio-temporal representation of categorical discrimination of emotion expressions in the human brain, i.e. the higher-order representation of emotions. However, the mental states that support mechanisms of emotion processing can be abstract and highly dimensional, rendering it difficult to disentangle which part of the processes linked to emotion discrimination are better conceptualized as categorical or dimensional as well as, which of these processes are driven by acoustic cues (see Giordano et al. 2021; Hamann 2012; Kragel and LaBar 2016; Skerry and Saxe 2015). Thus, to add to this knowledge gap, we propose a method that demonstrates categorical responses from the human brain to different discrete vocal emotions that are at least partially independent from processing of acoustic features. In the future, frequency tagging can also be used to investigate dimensional aspects of emotions such as valence, arousal, intensity etc. to unravel the interplay between categorical and felt perception of emotions and investigating the contribution of acoustic features to both processes. This makes frequency-tagging a valuable technique to study emotion categorization, suitable to use in populations that are more difficult to test (e.g., individuals with autism, infants etc.) with traditional paradigms such as ERP design studies and other neuroimaging techniques.

### Supplementary Information

Below is the link to the electronic supplementary material.

Supplementary file1 (DOCX 9 KB)

Supplementary file2 (PDF 39 KB)

Supplementary file3 (PDF 133 KB)

## Data Availability

The datasets generated during and/or analyzed during the current study are available from the corresponding author on reasonable request.
