# Supplementary figures and images for: Automatic Brain Categorization of Discrete Auditory Emotion Expressions
Source: Brain Topogr. 2023 Aug 28;36(6):854–69. doi: 10.1007/s10548-023-00983-8 (PMC10522533; doi:10.1007/s10548-023-00983-8)

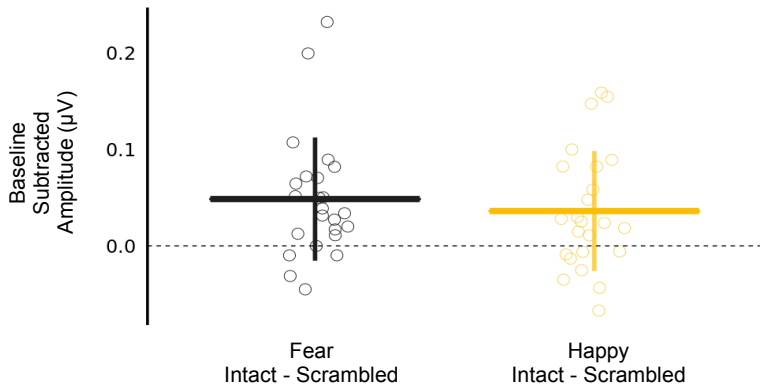

Supplement: Supplementary file 2 — Supplementary file2 (PDF 39 KB) [file 10548_2023_983_MOESM2_ESM.pdf]

Fear  
Intact - Scrambled

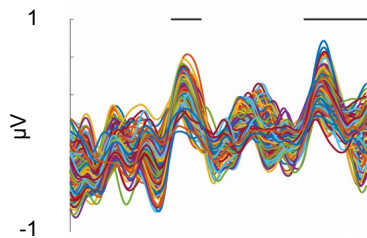

Happy  
Intact - Scrambled

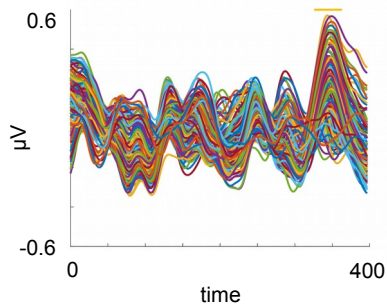

Supplement: Supplementary file 3 — Supplementary file3 (PDF 133 KB) [file 10548_2023_983_MOESM3_ESM.pdf]
